# 

**Published:** 1898-04

**Authors:** 


### CONTENTS.

COMMUNICATIONS.

The Influence and Power of Association .......... 151
Electricity in the Treatment of Pulpless Teeth .......... 157
The Methods of Using Gold and Tin Together .......... 166
A Clinic on a Fusible Alloy .......... 168
Some Causes of Failures in Operative Dentistry .......... 171
Instrument Nomenclature with Reference to Instrumentation .......... 174

PROCEEDINGS.

The Southern Branch of the National Dental Association .......... 191

SELECTIONS.

To Remove Nitrate of Silver Stains .......... 190
To Stop Nose-Bleeding .......... 190
A Newly Discovered Constituent of the Blood .......... 192
Physiological Testing of Drugs .......... 193
A Large Dussand Microphonograph .......... 194
Improved Local Anesthesia .......... 195
To Remove Color of Iodine and Permanganate of Potassium .......... 195
Tuberculosis of the Salivary Glands .......... 196
Alcohol as a Disinfectant .......... 196

EDITORIAL.

Tri-State Meeting .......... 196
The American Dental Weekly .......... 197
The Dental News .......... 198
Matrimonial .......... 198
The Eastern Indiana Dental Association .......... 198
The Indiana Dental Journal .......... 199
The Southern Branch of the National Dental Association .......... 199
Correction .......... 200
Bibliographical .......... 200
The National Dental Association—Division of the East .......... 200

